# Health cadres' experiences in detecting and preventing childhood stunting in Indonesia: a qualitative study

**DOI:** 10.1186/s12889-025-24192-z

**Published:** 2025-08-31

**Authors:** Sukmawati Sukmawati, Yanti Hermayanti, Eddy Fadlyana, Indra Maulana, Henny Suzana Mediani

**Affiliations:** 1 Doctoral Study Program, Faculty of Medicine, Universitas Padjadjaran, Bandung, West Java Indonesia; 2 Department of Maternity Nursing, Faculty of Nursing, Universitas Padjadjaran, Bandung, West Java Indonesia; 3 Department of Child Health, Faculty of Medicine, Universitas Padjadjaran, Bandung, West Java Indonesia; 4 Department of Mental Health Nursing, Faculty of Nursing, Universitas Padjadjaran, Bandung, West Java Indonesia; 5 Department of Pediatric Nursing, Faculty of Nursing, Universitas Padjadjaran, Bandung, West Java 45353 Indonesia

**Keywords:** Childhood, Detecting and preventing, Health cadres, Preventing, Stunting

## Abstract

**Background:**

Stunting in children is still a public health problem in Indonesia and concerns the Indonesian government. It can have both immediate and lasting impacts on human capital development and the future productivity of those affected individuals as they mature. Therefore, healthcare professionals and health cadres must prioritize detecting and preventing stunting in childhood within Indonesia’s healthcare context.

**Purpose:**

This study aims to understand and describe the experiences of health cadres in detecting and preventing stunting in childhood.

**Methods:**

A qualitative approach was used. From August to September 2023, 15 health cadres were interviewed individually, semi-structured, and in-depth. The data was analyzed using thematic analysis.

**Results:**

Four theme clusters emerged from the analysis: (1) challenges in detecting stunting in childhood, (2) effective communication with pregnant women, husbands and their families, (3) the need to increase knowledge in detecting and preventing stunting, (4) role and responsibility as health cadres in preventing stunting.

**Conclusion:**

The findings of this study provide valid primary data that can enhance the knowledge and skills of health cadres in detecting and preventing stunting in children. Training is necessary to improve volunteers’ basic knowledge and communication skills, supporting early awareness and family engagement in stunting prevention among children in Indonesia.

**Supplementary Information:**

The online version contains supplementary material available at 10.1186/s12889-025-24192-z.

## Introduction

Stunting poses a direct challenge to achieving the Sustainable Development Goals (SDGs), especially SDG 2: Zero Hunger, which aims to combat malnutrition and ensure consistent access to nutritious food. One of its key targets is to halve the prevalence of stunting among children by 2030 [4].

Inadequate nutrition during the first 1,000 days of life, from conception to a child’s second birthday, is a primary cause of stunting [5–7]. In addition to inadequate nutrition, stunting can result from chronic or recurrent infections and anemia. Various contributing factors include household and family-related conditions, such as child-specific factors (low birth weight, premature birth), maternal characteristics (young maternal age, low education level, short stature, and large family size), infections, and inadequate breastfeeding practices [8]. Stunting is also closely linked to low socio-economic status and inadequate sanitation conditions [9]. Stunting may also stem from various factors, such as ineffective parenting practices, limited access to quality antenatal (ANC) and postnatal care (PNC), inadequate early childhood education services, insufficient maternal knowledge regarding nutritional needs before, during, and after pregnancy, and continued limited access to nutritious food and clean water [10, 11]. 

Stunting adversely affects children’s cognitive, motor, and language development, leading to increased risks of illness and mortality. As they grow into adulthood, they often exhibit shorter stature and tend to have lower learning capacity and academic performance during their school years [11, 12]. In the short term, stunting can lead to adverse effects, including impaired brain development, reduced intellectual capacity, physical growth delays, and metabolic disorders [13]. In the long term, stunting can result in diminished cognitive abilities and academic performance, weakened immunity, greater susceptibility to illness, and a heightened risk of chronic conditions such as diabetes, obesity, cardiovascular disease, cancer, stroke, and disabilities in later life [11]. According to the World Health Organization (2018), stunting is estimated to contribute to approximately one million child deaths annually [14]. 

In Indonesia, stunting has been a national priority. To address stunting, the Indonesian government issued Presidential Regulation No. 72 of 2021, which aims to accelerate the Reduction of Stunting. This regulation aims to promote the development of healthy, intelligent, and productive human resources while supporting the achievement of the United Nations’ Sustainable Development Goals (SDGs). It promotes a comprehensive, integrated, and high-quality approach to reducing stunting through coordinated, synergistic, and synchronized efforts among ministries and agencies, provincial and local governments, village administrations, and various stakeholders [15]. However, the stunting rate has not decreased significantly and remains below the WHO standard of 20%. This is due to parents’ limited knowledge, attitudes, and behaviors, as well as the support of families and health cadres in preventing stunting during the first 1000 days of life [16]. 

Efforts to prevent and detect stunting early have increasingly emphasized the critical role of community-based actors, including health cadres or community health workers [16]. Globally, these cadres are recognized as vital in bridging the gap between communities and health systems, particularly in underserved areas. Their responsibilities often include growth monitoring, nutrition counseling, promoting exclusive breastfeeding, complementary feeding education, and referring at-risk children to health facilities [17]. In several countries, including India, Ethiopia, and Indonesia, they play a crucial role in the early detection of growth faltering and delivering culturally appropriate, grassroots-level interventions [18–20]. 

Recognizing stunting as a national priority has further elevated the visibility and strategic involvement of community-based actors in prevention and intervention efforts. In efforts to prevent and detect stunting early in Indonesia, the government has launched an integrated strategy that involves multisectoral collaboration, including health cadres as key implementers at the community level. Minister of Health Regulation Number 19 of 2017 emphasizes the importance of healthcare personnel and cadres in assisting maternal and child health services as an initial measure to prevent stunting. These cadres assist with monthly growth monitoring, provide nutrition education to mothers, and facilitate access to maternal and child health services. Their unique position within communities makes their experiences, insights, and challenges especially valuable in understanding the local context of stunting prevention and early detection. Additionally, the involvement of health cadres in stunting prevention efforts is further underscored by the Ministry of Villages for Development of Disadvantaged Regions Regulation Number 19 of 2017, particularly in point 9, which emphasizes community empowerment to enhance overall health and well-being. The skills and capabilities of health cadres can help implement targeted interventions to identify and address stunting risks among pregnant women [11]. Health cadres also play a crucial role in tasks such as collecting data on toddlers, recording their weight on Health Cards, providing supplementary food, offering nutritional education, distributing vitamins, conducting home visits to nursing and pregnant mothers with toddlers, and measuring height, which is essential for detecting cases of stunting [21]. 

The proactive involvement of health cadres in the early prevention of stunting within Integrated health centres plays an essential role in enhancing public health quality. Early intervention for stunting prevention has proven to be an effective strategy for reducing its prevalence. As frontline representatives within the Integrated Health Center, health cadres are crucial in implementing impactful interventions to reduce stunting rates among children. Knowledge, experience, and skills can help prepare health cadres to maximize their effectiveness and contribute significantly to this effort. Utilizing their expertise and strong community connections, health workers can implement targeted interventions, educational initiatives, and support programs to identify and address stunting risk factors early. Through active engagement and continuous improvement efforts, health cadres play a crucial role in fostering healthier outcomes for children and communities [14, 21]. The role of health cadres in preventing stunting is crucial. They support the early detection and prevention of childhood stunting by facilitating health education, encouraging regular maternal and child health check-ups, and promoting family awareness [16]. 

According to prior research, many healthcare professionals are proficient in identifying and preventing stunting risks [22, 23]. Other research indicates that empowering cadres at integrated health service posts, particularly concerning optimizing the First 1,000 Days of Life, enhances their leadership role in these efforts. It also contributes to improving their knowledge and skills in preventing stunting [24, 25]. Nevertheless, the active engagement of health cadres in these endeavors appears to vary based on age, education, employment status, and tenure [16]. This variance is primarily attributed to internal factors, notably the experiences and perceptions of health cadres regarding stunting detection and prevention efforts. Certain cadres may exhibit less proactive involvement, despite having adequate knowledge, due to differing experience levels, personal beliefs, and contextual challenges in their work environments. These internal influences underscore the importance of addressing knowledge gaps and fostering a supportive environment that encourages and empowers health cadres to play a more active role in stunting prevention initiatives. By addressing these internal factors and providing targeted support and training, health cadres can be further empowered to effectively reduce stunting prevalence and promote healthier outcomes within their communities [16]. 

Despite their importance, limited qualitative research explores the lived experiences of health cadres in detecting and preventing stunting in Indonesia. Understanding their perspectives is crucial for strengthening community-based interventions, informing policy, and improving support systems for these frontline workers. This study aims to explore the experiences of health cadres in Indonesia, shedding light on their roles, challenges, motivations, and contributions in the fight against childhood stunting. This study aimed to explore and elucidate the experiences of health cadres in identifying and mitigating stunting risks among children within the Indonesian context.

## Materials and methods

### Design

This study employs a qualitative research design to investigate the experiences of health cadres regarding the detection and prevention of stunting among children in Indonesia. Qualitative research, known for its exploratory nature, offers an approach to comprehensively understanding the meanings attributed by individuals or groups in response to social or human issues [26]. 

### Setting and participants

This research was conducted at the Garut Regency Health Center in West Java Province, which is known for having the highest incidence of stunting in its region. Participants were recruited through direct engagement facilitated by health personnel at *posyandu* (Integrated Health Service Post) and community health centre, based on recommendations from the head of the designated work area within the study setting.

This study employed purposive sampling to deliberately select individuals with relevant experience and insights related to the detection and prevention of stunting. Purposive sampling is appropriate for qualitative research, as it aims to explore specific experiences in depth rather than to generalize findings. Purposive sampling helped increase credibility by enabling a deeper exploration of participants’ experiences, but these findings cannot necessarily be generalized to other contexts. This is a known issue in qualitative research, which emphasizes context-based knowledge building rather than statistical generalization. Therefore, this sampling strategy is suitable for qualitative studies that aim to explore specific experiences in-depth, rather than generalizing results, and does not introduce bias; instead, it enhances the credibility and relevance of the data by focusing on participants who are most knowledgeable about the phenomenon under investigation. Eligible participants included community health volunteers who had been actively engaged for at least six months, were 15 years or older, and were directly involved in supporting pregnant women. Volunteers who had not participated in community health activities within the past three months or who declined to provide written informed consent were excluded from the study.

#### Data collection

Data was collected through in-depth, semi-structured interviews conducted in person at a location convenient to the participants. An interview guide explored participants’ roles, experiences, challenges, and perspectives on stunting prevention and early detection. All interviews were audio-recorded with participants’ consent and transcribed verbatim in Indonesian.

Semi-structured in-depth interviews comprising questions aimed at eliciting participants’ perceptions and experiences related to detecting and preventing stunting in children. Unlike survey-based research, qualitative studies typically involve a smaller number of participants. In-depth interviews utilizing semi-structured questions do not have a predetermined minimum number of respondents. For this type of research, the recommended sample size ranges from 5 to 25 participants, ensuring a rich and comprehensive exploration of the research topic [27]. In the descriptive information section, data were collected regarding participants’ age, gender, education, occupation, and tenure as a health cadre. The overarching research question guiding this study was: “What are the experiences of health cadres in detecting and preventing stunting in Childhood?”

The data collection process took place in October 2023. Initially, the researcher gathered the participants’ contact details and scheduled interview dates according to their preferences. According to the schedule, health cadres were invited to face-to-face interviews. These interviews were conducted at each participant’s home to minimize interference with their other responsibilities. Before commencing the interview, the researcher explained the study’s purpose and assured participants of their right to withdraw from the study at any time if they felt uncomfortable.

Additionally, the confidentiality of the information shared was emphasized. Each participant is anonymous and assigned a random code. Participants were granted permission to record the interviews to facilitate accurate monitoring of their feedback and responses. Each interview was attended by two researchers, with one serving as the interviewer and the other as the note-taker. Participants were encouraged to provide clear interpretations and seek further clarification if any ambiguity arose during the interviews. The interviews were conducted predominantly in Indonesian. Each interview averages between 30 and 40 min. Subsequently, two participants were asked to review the list of questions, with no modifications made to the interview questions based on their feedback.

Data collection continued until data saturation was reached, when no new themes or insights emerged from subsequent interviews. Saturation was assessed concurrently with data collection through iterative review of transcripts and preliminary coding. When later interviews consistently confirmed previously identified patterns without adding novel information, the research team determined that data saturation had been achieved and ceased further recruitment.

#### Data analysis

Data were analyzed using a thematic analysis approach, following the six-phase framework developed by Braun and Clarke [28]. Firstly, the researchers familiarized themselves with the data by repeatedly reading the verbatim transcripts. Next, open coding was conducted to identify meaningful units of text, which were then grouped into sub-themes based on conceptual similarities. These sub-themes were further reviewed and synthesized into overarching themes. The emerging themes were critically examined and refined to ensure coherence with the data. Finally, each theme was clearly defined and labeled to capture its essence. Coding was conducted manually and discussed collaboratively within the research team to enhance analytical accuracy and ensure dependability.

#### Rigour and trustworthiness of the study

The analysis adhered to Lincoln and Guba’s four evaluative criteria to ensure the study’s rigour and trustworthiness [29]. Credibility was established through member checking, whereby selected participants reviewed and confirmed the accuracy of the findings. Dependability was supported by maintaining an audit trail documenting key decisions and analytical steps throughout the research process. Confirmability was achieved through regular team debriefings and reflexive journaling to minimize research bias. Finally, transferability was addressed by providing rich, detailed descriptions of the study context, participants, and thematic findings, allowing readers to assess the applicability of results to other settings.

## Results

### Characteristics of participants

The qualitative study involved 15 health cadres from a Community Health Center with high stunting rates who met the eligibility criteria or expressed willingness to participate. The average age of all female and Muslim participants was 28.4 years. Most (60%) had completed high school, and most (86.7%) were homemakers. On average, they had been serving as cadres for 8.7 years. All participants were actively engaged in efforts to detect and prevent stunting. These demographic details are crucial for understanding the perspectives and experiences of health cadres regarding stunting detection and prevention. A comprehensive overview of the participants’ characteristics is presented in Table 1.

**Table 1:** Characteristics of participants (*n* = 15)

Number	Age (year)	Biological Gender	Religion	Education	Occupation	Length of time working as a health cadre (year)
P1	39	Female	Islam	Senior High School	Housewife	3
P2	28	Female	Islam	Senior High School	Housewife	29
P3	51	Female	Islam	Junior high school	Housewife	23
P4	46	Female	Islam	Junior high school	Housewife	15
P5	47	Female	Islam	Senior High School	Housewife	1
P6	39	Female	Islam	Senior High School	Housewife	3
P7	46	Female	Islam	College	Private sector employee	8
P8	49	Female	Islam	Senior High School	Housewife	10
P9	58	Female	Islam	Elementary school	Housewife	2
P10	49	Female	Islam	Senior High School	Housewife	15
P11	45	Female	Islam	Elementary school	Housewife	1
P12	28	Female	Islam	Senior High School	Private sector employee	4
P13	28	Female	Islam	Senior High School	Housewife	5
P14	34	Female	Islam	Elementary school	Housewife	5
P15	37	Female	Islam	Senior High School	Housewife	5

### Study outcome

The study identified four primary themes: (1) challenges in identifying stunting among pregnant women, (2) strategies for effective communication with pregnant women and their families, (3) the necessity of enhancing knowledge regarding stunting detection and prevention, and (4) the roles and duties of health cadres in stunting prevention. The first theme reveals the barriers faced by health cadres, such as a lack of support from husbands and low participation of pregnant women in antenatal care services. The second theme emphasizes the importance of effective communication, not only with pregnant women but also with their families, particularly their husbands, to create a supportive environment. The third theme highlights the crucial need to enhance the knowledge of health cadres, enabling them to identify and prevent stunting through early detection and education more effectively. Lastly, the fourth theme highlights the multifaceted roles of health cadres as educators, counselors, and facilitators, who are instrumental in community-based initiatives aimed at preventing stunting. Further details, including overarching themes, subthemes, and illustrative quotations, are presented in Table 2. This qualitative research highlights the experiences of health cadres in detecting and preventing stunted growth in children.

**Table 2:** Superordinate, subordinate themes, and participant quotes

Superordinate Themes	Subordinate Themes	Quotes
Challenges in identifying stunting among pregnant women	Lack of support from husband and family	Q1: “Health cadres frequently emphasize to pregnant women the importance of regularly attending the Posyandu to monitor their fetuses’ growth and development to prevent stunting. However, some pregnant women refrain from attending because their husbands prohibit them.” (P10) Q2: “I continue to sense a lack of complete support from the families of pregnant women regarding the detection of stunting risks, as they often attribute stunted growth in children to hereditary factors, considering it a common occurrence.” (P7) Q3: “Some pregnant women still resist undergoing pregnancy checks at integrated service posts”. (P5).
	Lack of participation of pregnant women	Q4: “Several mothers still decline to undergo pregnancy checks for various reasons”. (P3) Q5: “Despite receiving health education on stunting detection, some pregnant women still exhibit limited responsiveness. (P9)
Strategies for effective communication with pregnant women, husbands, and their families	Communication with pregnant women	Q6: ”For successful risk detection and prevention of stunting, effective communication with pregnant women is crucial, helping them recognize the significance of preventing stunting in their fetus.” (P15) Q7: “I endeavour to foster positive communication with pregnant women, encouraging their willingness to undergo early detection and take steps to prevent stunting, such as consuming nutritious food.”. (P1)
	Communication with their family	Q8: “I try to tell families to work together to prevent stunting in pregnant women. (P11) Q9: “For prevention efforts against stunting to be effective, the involvement of families is essential throughout their implementation”. (P6) Q10: “Establishing effective communication with families, particularly husbands, is crucial to garnering support for the detection and prevention of stunting in pregnant women.” (P12)
The necessity of enhancing knowledge regarding the detection and prevention of stunting	Knowledge Detecting stunting	Q11: “Health cadres require empowerment to aid in the detection and prevention of stunting, given their proximity to pregnant women.” (P8) Q12: “Health cadres frequently encourage pregnant women to attend integrated service posts regularly to monitor the growth and development of their fetuses, thereby reducing the risk of stunting” (P5). Q13: “Health cadres must enhance their knowledge about detecting the risk of stunting so they can assist pregnant women in identifying such risks in their fetuses.”. (P13)
	Knowledge Preventing Stunting	Q14: “Not all health cadres are equipped with the knowledge of stunting prevention. Therefore, it’s essential to provide refresher training sessions for health cadres to enhance their understanding of stunting prevention."(P14) Q15: “Health cadres must undergo training to enhance their knowledge and capacity to contribute to stunting prevention efforts” (P2) effectively Q16: “Health cadres with good knowledge about stunting prevention will find it easier to help pregnant women prevent stunting”. (P15)
The roles and duties of health cadres in stunting prevention	Role health cadres in preventing stunting	Q17: “Health cadres play an essential role in preventing stunting since they are the individuals closest to pregnant women”. (P8) Q18: “The role of health cadres as educators, counsellors and companions for pregnant women in risk detection and prevention of stunted, because cadres are the people closest to pregnant women and their families.“. (P10)
	Duties of health cadres in preventing stunting	Q19: “Providing counselling to pregnant women to prevent stunting in my duties as health cadre. Therefore, I often provide counselling while chatting casually so that pregnant women understand (P4) Q20: “Stunting prevention is a joint duty of the government, families and all levels of society, so implementation must be carried out collaboratively to achieve optimal results”. (P1)

## Discussion

This research shows the importance of stunting detection and prevention involving health cadres, the need for health cadres to be able to overcome the challenges of lack of support and understanding of pregnant women, husbands, and their families, increase effective communication with pregnant women, husbands, and their families, increase knowledge, play an optimal role with high dedication in carrying out detection and prevention of childhood stunting.

### Challenges in identifying stunting among pregnant women

Early detection of stunting requires regular monitoring of risk factors that affect fetal growth and development during pregnancy and in toddlers. Consistent monitoring of these risk factors in both the womb and children under five is essential for early detection of stunting.

Pregnant women and children under five should receive regular health check-ups, accessible through integrated health centres in their communities. Health cadres are crucial in encouraging pregnant women and mothers to attend these check-ups, facilitating early detection and prevention efforts for stunting. It’s emphasized that prevention efforts should commence as early as possible for optimal results, necessitating behavioural changes in pregnant women due to the significance of stunting prevention [30]. 

The study findings highlight several challenges faced by health cadres in identifying pregnant women about the risk of stunting in the fetus they are carrying. These challenges include a lack of understanding among pregnant women about stunting and its prevention, as well as the factors contributing to stunting in childhood. Pregnant women and breastfeeding mothers lack awareness of the importance of proper child care and nutritious food consumption in optimizing maternal and child health to prevent stunting. Due to the digital divide and limited internet access in rural areas, health cadres lack access to Community-Based Nutrition Recording and Reporting applications.

Health cadres are responsible for assessing child growth and development, detecting stunting in pregnant women, and monitoring nutritional status using Maternal and Child Health Cards. However, there are instances where health cadres, particularly in remote areas, inaccurately record data from weighing scales and height measurements, leading to biased recording and subsequent misinterpretation and mismanagement of stunting cases. Additionally, challenges are exacerbated when health cadres lack access to the Community-Based Nutrition Recording and Reporting application due to the digital divide and limited internet accessibility in rural regions [31]. 

Another challenge faced by health cadres in identifying and preventing stunting is the lack of knowledge among mothers and the low participation of families and communities in identifying and preventing stunting [32]. Participation from mothers and families is essential in efforts to detect and avoid stunting through effective parenting practices. Parenting patterns in the detection and prevention of stunting are the way parents care by paying attention to nutrition and health from the time the fetus is in the womb, educating and caring for children, and providing good stimulation so that the child’s growth and development is optimal, mothers and families always accompany their children and pay attention, especially in provide food intake which contains good nutrition, so that it is hoped that children will have good nutritional status and avoid stunting [33]. One factor contributing to stunting is low public awareness and behavior due to a lack of understanding of health [34]. Support from various stakeholders, including healthcare workers, is necessary to increase public awareness, particularly among pregnant women and mothers with children under five. A previous study by Wulandari and Kusumastuti shows that the support of health workers is crucial in preventing and overcoming stunting [32]. 

### Strategies for effective communication with pregnant women, husbands, and their families

Effective communication with pregnant women, their partners, and their families is crucial. Health cadres who struggle to communicate with the community, especially with pregnant women, husbands, and their families, often find it challenging to prevent stunting effectively. Conversely, proficient communication skills enable health cadres to implement stunting prevention efforts effectively. Health cadres must provide a comprehensive understanding to pregnant women regarding the risks associated with pregnancy, encourage regular antenatal care visits, and impart knowledge about pregnancy, childbirth, postpartum care, and, importantly, stunting prevention. Communication and counselling efforts by health cadres extend beyond pregnant women to include their families, such as spouses, parents, and other close individuals, who can provide crucial support and supervision throughout the pregnancy journey. Communication supports the success of intervention programs aimed at individuals, groups, and society [34, 35]. 

The quality of communication with pregnant women and mothers of stunted children under the age of five directly influences the successful fulfilment of nutritional requirements, serving as a crucial aspect of stunting prevention efforts. Families play a pivotal role in ensuring that pregnant women and young children receive a sufficient dietary intake. They are tasked with making vital decisions regarding caregiving and providing nutritious meals. To facilitate this, families may need to adjust their roles formally and informally, particularly in cases where mothers do not engage in formal employment. This adjustment gives mothers the time to prepare meals with high nutritional value [36]. Consequently, effective communication between health cadres, pregnant women, and their families becomes indispensable.

### The necessity of enhancing knowledge regarding the detection and prevention of stunting

Health cadres encounter various challenges in identifying and preventing stunting, including the lack of awareness among pregnant women regarding the risks and prevention methods associated with stunting. A primary problem encountered by health cadres is their insufficient understanding of identifying indicators of stunting risk in pregnant mothers.

One potential solution to this problem is to leverage the role of health cadres. As pivotal figures in disseminating information and education to the public, particularly to pregnant women, health cadres can play a crucial role in stunting detection and prevention efforts. Competency-based training can be implemented immediately, utilizing visual modules and hands-on practical sessions to address this. Furthermore, continuous support from healthcare professionals through regular oversight can enhance the confidence and competencies of volunteers. This intervention enhances volunteer capacity and strengthens the community’s early detection system. Another strategy is Competency-based training, which can be implemented immediately by utilizing visual modules and practical hands-on sessions to address this issue. Furthermore, continuous support from healthcare professionals through regular oversight can enhance the confidence and competencies of volunteers. This intervention develops volunteer capacity and fortifies the early detection system within the community. Therefore, there is a pressing need to enhance the knowledge of health cadres in this regard. By equipping health cadres with adequate knowledge and awareness, it is anticipated that stronger collaboration can be fostered between healthcare professionals and the community, particularly pregnant women, in identifying and preventing stunting. This collaboration is expected to yield positive outcomes for children’s health and overall quality of life while raising awareness about the significance of optimal nutrition from early childhood. Health education targeted at health cadres emerges as an effective strategy in combating stunting [37]. 

Health cadres are crucial in advancing public health, particularly in addressing childhood stunting [38]. Thorough and suitable health education is expected to raise public awareness and understanding of stunting, while improving children’s well-being and overall quality of life [16]. One effective strategy to enhance the proficiency of health cadres in identifying and preventing stunting is providing health education [39]. 

Cultural factors also play a significant role in shaping the competencies and effectiveness of health cadres in detecting and preventing stunting. In many communities, deeply rooted beliefs about pregnancy, child-rearing practices, and traditional food taboos may hinder the acceptance of health messages promoted by cadres. For example, some pregnant women may avoid certain nutritious foods due to cultural myths or rely more heavily on traditional birth attendants than on formal healthcare services. These cultural norms can pose challenges for cadres attempting to promote behavior change. Additionally, in some regions, the influence of patriarchal norms may limit a woman’s autonomy in making health decisions, thereby requiring cadres to engage not only mothers but also husbands and elder family members in stunting prevention efforts. As such, improving the competency of health cadres must also involve enhancing their cultural sensitivity and communication strategies to navigate these complex social dynamics. Tailored training that incorporates culturally informed approaches may improve its effectiveness in addressing community-specific barriers and fostering greater community trust and cooperation.

### The roles and duties of health cadres in stunting prevention

The findings indicate that while health cadres may not engage in clinical diagnosis of stunting, they perceive their role as instrumental in its early detection and prevention. Their involvement in stunting-related educational campaigns, family counseling, and outreach efforts, particularly targeting pregnant women and families, positions them as critical agents of community awareness. These actions, informed by ongoing training and local health initiatives, provide a supportive environment where families are better informed and more responsive to their child’s health and nutritional needs. Thus, their perceived responsibility in stunting prevention reflects an active contribution to public health strategies at the grassroots level. Their involvement positively impacts garnering family support for detecting and preventing stunting. This support is facilitated through educational initiatives, training programs, and awareness-raising efforts. In promoting healthier lifestyles and combating stunting, health cadres play a crucial role in imparting education and fostering awareness within the community, particularly among pregnant women. Their pivotal role in stunting prevention stems from their frontline position in disseminating information and supporting the community, especially pregnant women, regarding nutrition and child health [40]. Health cadres’ duties in preventing stunting include providing additional food, distributing vitamin A, offering nutritional education to pregnant women, visiting homes, and serving as community health educators, particularly among pregnant women and their families [41]. 

The role of cadres has a positive influence on family support in preventing and overcoming stunting [32]. Healthcare cadres are essential in enhancing the community’s ability to help themselves achieve optimal health and develop their health sector. Health cadres must ensure that all necessary preventive and protective measures are taken to minimize safety and health risks, including preventing disorders.

The findings of this study highlight the critical role of community health volunteers in encouraging pregnant women to utilize maternal health services. This aligns with previous research from Bangladesh, which demonstrated that health cadres effectively increased antenatal care (ANC) attendance among expectant mothers [40]. However, in contrast to these earlier studies, our research uncovered a substantial gap in the competencies of Indonesian health volunteers, particularly in identifying early indicators of stunting risk during pregnancy. Interview data revealed notable knowledge gaps, with health cadre participants acknowledging that the absence of targeted training in stunting detection and prevention, along with challenges such as inaccurate data recording at community health centers, posed significant barriers to effective intervention. This highlights the need for more structured and comprehensive training focused on early detection strategies. These insights contribute to a deeper understanding of the importance of empowering community-based volunteers as facilitators of healthcare access and pivotal actors in the early prevention of stunting. Strengthening their capacity may enhance the effectiveness of community-based interventions and support national efforts to reduce stunting prevalence from the earliest stages of the maternal-child health continuum.

## Conclusion

This qualitative study examined the experiences of health cadres in supporting the early identification and prevention of childhood stunting in Indonesia. The findings highlight that while health cadres contribute significantly to community-based stunting prevention, particularly through their outreach at Integrated Service Posts, they encounter several challenges, including limited family support (especially from husbands) and low awareness among pregnant women about the risks of stunting. Cadres should be equipped with targeted training and practical knowledge to optimize their role, especially in recognizing early signs of child growth issues and promoting maternal health-seeking behavior. Enhancing their communication and health promotion skills is also crucial for effectively engaging mothers and families as active partners in prevention efforts. As trusted community members, cadres can serve as facilitators and advocates within their local context, helping bridge communities with the health system and support national strategies to reduce stunting.

### Implications of the study

The findings of this study carry several important implications for public health policy, community-based health programs, and future research. First, the study emphasizes the importance of ongoing, context-specific training to enhance the capacity of health cadres to support early stunting prevention efforts, particularly by recognizing early risk indicators during pregnancy and promoting timely utilization of health services. Providing cadres with evidence-informed knowledge and practical communication skills can strengthen their role as facilitators of health education and early awareness at the community level. Second, the findings underscore the crucial role of family engagement, particularly husbands’ involvement, in supporting maternal and child health initiatives. Public health strategies should incorporate family-centered communication approaches that empower cadres to mobilize broader family support for stunting prevention. Third, this study supports the formal integration of health cadres into maternal and child health policies and programs. Recognizing their unique position as trusted community figures, policy frameworks should ensure adequate support through structured training, supervision, incentives, and resources to sustain and enhance their effectiveness. Ultimately, this research highlights the importance of incorporating community voices into implementing public health interventions. Future research should focus on evaluating scalable models for health cadres training and support and assessing the measurable impact of their engagement on stunting-related outcomes.

## Supplementary Information

Supplementary Material 1.

Supplementary Material 2.

## Data Availability

The datasets generated and analysed during the current study are available from the corresponding author on reasonable request.
