# Supplementary material for: Health cadres' experiences in detecting and preventing childhood stunting in Indonesia: a qualitative study
Source: BMC Public Health. 2025 Aug 31;25:2987. doi: 10.1186/s12889-025-24192-z (PMC12400572; doi:10.1186/s12889-025-24192-z)
Supplement: Supplementary file 1 — Supplementary Material 1. [file 12889_2025_24192_MOESM1_ESM.docx]

**Table 1** **Characteristics of Participants (n=15)**

| Number | Age (year) | Biological Gender | Religion | Education | Occupation | Length of time working as a health cadre (year) |
| --- | --- | --- | --- | --- | --- | --- |
| P1 | 39 | Female | Islam | Senior High School | Housewife | 3 |
| P2 | 28 | Female | Islam | Senior High School | Housewife | 29 |
| P3 | 51 | Female | Islam | Junior high school | Housewife | 23 |
| P4 | 46 | Female | Islam | Junior high school | Housewife | 15 |
| P5 | 47 | Female | Islam | Senior High School | Housewife | 1 |
| P6 | 39 | Female | Islam | Senior High School | Housewife | 3 |
| P7 | 46 | Female | Islam | College | Private sector employee | 8 |
| P8 | 49 | Female | Islam | Senior High School | Housewife | 10 |
| P9 | 58 | Female | Islam | Elementary school | Housewife | 2 |
| P10 | 49 | Female | Islam | Senior High School | Housewife | 15 |
| P11 | 45 | Female | Islam | Elementary school | Housewife | 1 |
| P12 | 28 | Female | Islam | Senior High School | Private sector employee | 4 |
| P13 | 28 | Female | Islam | Senior High School | Housewife | 5 |
| P14 | 34 | Female | Islam | Elementary school | Housewife | 5 |
| P15 | 37 | Female | Islam | Senior High School | Housewife | 5 |
