# Supplementary material for: Health cadres' experiences in detecting and preventing childhood stunting in Indonesia: a qualitative study
Source: BMC Public Health. 2025 Aug 31;25:2987. doi: 10.1186/s12889-025-24192-z (PMC12400572; doi:10.1186/s12889-025-24192-z)
Supplement: Supplementary file 2 — Supplementary Material 2. [file 12889_2025_24192_MOESM2_ESM.docx]

**Table 2: Superordinate, Subordinate Themes, and Participant Quotes**

| Superordinate Themes | Subordinate Themes | Quotes |
| --- | --- | --- |
| Challenges in identifying stunting among pregnant women | Lack of support from husband and family | Q1: “Health cadres frequently emphasize to pregnant women the importance of regularly attending the Posyandu to monitor their fetuses' growth and development to prevent stunting. However, some pregnant women refrain from attending because their husbands prohibit them.” (P10)  Q2: “I continue to sense a lack of complete support from the families of pregnant women regarding the detection of stunting risks, as they often attribute stunted growth in children to hereditary factors, considering it a common occurrence.” (P7)  Q3: “Some pregnant women still resist undergoing pregnancy checks at integrated service posts”. (P5). |
|  | Lack of participation of pregnant women | Q4: “Several mothers still decline to undergo pregnancy checks for various reasons”. (P3)  Q5: “Despite receiving health education on stunting detection, some pregnant women still exhibit limited responsiveness. (P9) |
| Strategies for effective communication with pregnant women, husbands, and their families | Communication with pregnant women | Q6: ”For successful risk detection and prevention of stunting, effective communication with pregnant women is crucial, helping them recognize the significance of preventing stunting in their fetus.” (P15)  Q7: “I endeavour to foster positive communication with pregnant women, encouraging their willingness to undergo early detection and take steps to prevent stunting, such as consuming nutritious food.”. (P1) |
|  | Communication with their family | Q8: “I try to tell families to work together to prevent stunting in pregnant women. (P11)  Q9: “For prevention efforts against stunting to be effective, the involvement of families is essential throughout their implementation”. (P6)  Q10: “Establishing effective communication with families, particularly husbands, is crucial to garnering support for the detection and prevention of stunting in pregnant women.” (P12) |
| The necessity of enhancing knowledge regarding the detection and prevention of stunting | Knowledge Detecting stunting | Q11: “Health cadres require empowerment to aid in the detection and prevention of stunting, given their proximity to pregnant women.” (P8)  Q12: “Health cadres frequently encourage pregnant women to attend integrated service posts regularly to monitor the growth and development of their fetuses, thereby reducing the risk of stunting” (P5).  Q13: “Health cadres must enhance their knowledge about detecting the risk of stunting so they can assist pregnant women in identifying such risks in their fetuses..”. (P13) |
|  | Knowledge Preventing Stunting | Q14: “Not all health cadres are equipped with the knowledge of stunting prevention. Therefore, it's essential to provide refresher training sessions for health cadres to enhance their understanding of stunting prevention. " (P14)  Q15: “Health cadres must undergo training to enhance their knowledge and capacity to contribute to stunting prevention efforts” (P2) effectively  Q16: “Health cadres with good knowledge about stunting prevention will find it easier to help pregnant women prevent stunting”. (P15) |
| The roles and duties of health cadres in stunting prevention | Role health cadres in preventing stunting | Q17: “Health cadres play an essential role in preventing stunting since they are the individuals closest to pregnant women”. (P8)  Q18: “The role of health cadres as educators, counsellors and companions for pregnant women in risk detection and prevention of stunted, because cadres are the people closest to pregnant women and their families.". (P10) |
|  | Duties of health cadres in preventing stunting | Q19: "Providing counselling to pregnant women to prevent stunting in my duties as health cadre. Therefore, I often provide counselling while chatting casually so that pregnant women understand (P4)  Q20: "Stunting prevention is a joint duty of the government, families and all levels of society, so implementation must be carried out collaboratively to achieve optimal results”. (P1) |
